# Enhancing the weighted voting ensemble algorithm for tuberculosis predictive diagnosis

**DOI:** 10.1038/s41598-021-94347-6

**Published:** 2021-07-20

**Authors:** Victor Chukwudi Osamor, Adaugo Fiona Okezie

**Affiliations:** Department of Computer and Information Sciences, College of Science and Technology (CST), Covenant University, Ota, Ogun State Nigeria

**Keywords:** Computational biology and bioinformatics, Molecular biology, Systems biology, Biomarkers, Diseases, Health care, Mathematics and computing

## Abstract

Tuberculosis has the most considerable death rate among diseases caused by a single micro-organism type. The disease is a significant issue for most third-world countries due to poor diagnosis and treatment potentials. Early diagnosis of tuberculosis is the most effective way of managing the disease in patients to reduce the mortality rate of the infection. Despite several methods that exist in diagnosing tuberculosis, the limitations ranging from the cost in carrying out the test to the time taken to obtain the results have hindered early diagnosis of the disease. This work aims to develop a predictive model that would help in the diagnosis of TB using an extended weighted voting ensemble method. The method used to carry out this research involved analyzing tuberculosis gene expression data obtained from GEO (Transcript Expression Omnibus) database and developing a classification model to aid tuberculosis diagnosis. A classifier combination of Naïve Bayes (NB), and Support Vector Machine (SVM) was used to develop the classification model. The weighted voting ensemble technique was used to improve the classification model's performance by combining the classification results of the single classifier and selecting the group with the highest vote based on the weights given to the single classifiers. Experimental analysis indicates a performance accuracy of the enhanced ensemble classifier as 0.95, which showed a better performance than the single classifiers, which had 0.92, and 0.87 obtained from SVM and NB, respectively. The developed model can also assist health practitioners in the timely diagnosis of tuberculosis, which would reduce the mortality rate caused by the disease, especially in developing countries.

## Introduction

Tuberculosis (TB) has a maximum level of mortality caused by a single micro-organism when compared to other diseases. Tuberculosis, therefore, is a significant health issue worldwide and a vital issue for most developing countries due to low treatment and diagnosis methods^1–3^. As indicated in World Health Organization (WHO) report in 2015, TB is the world most prevalent cause of death because it is an infectious disease, with 10 million individuals affected and 1.8 million deaths reported in total^4^. Approximately 10 million people were diagnosed with TB in 2018, and 1.6 million people died of TB in total. In 2018, this disease was also one of the world's top ten death reasons^5^. Tuberculosis (TB) is a frequently fatal, common, infectious, and endemic bacterial disease caused by Mycobacterium in humans, called Mycobacterium tuberculosis. It usually passes through the air and affects patients with chronic diseases and weak immune systems, such as diabetes, Human Immunodeficiency Virus (HIV), chronic kidney disease, and silicosis. Long-term malnutrition and alcohol misuse are independent disease risk factors, including ages under four years^2,6^. Tuberculosis is a disease that can damage almost all organs, not excluding even those locations that are comparatively inaccessible. Typically, micro-organisms enter the body by inhalation through the lungs. They travel from the initial position in the lungs via the bloodstream to other parts of the body. They pose a diagnostic challenge even for doctors with extensive knowledge of the disease^2^, people who are not appropriately treated are more likely to have multidrug-resistant tuberculosis^3^. Early diagnosis and evaluation of the TB severity stage are essential for determining the right treatment and eventually avoiding the death of curable patients’ cases^5^. If the bacteria that causes TB enters the body the following three things may happen: body kills bacteria, so there is no harm, the bacteria remain silent in the human body, this is called "Latent TB" or the body becomes ill due to bacteria inherent in the human system and is called "Active TB"^7^.

Despite the several methods that exist in the diagnosis of tuberculosis, the limitations ranging from the cost in carrying out the test to the time taken to obtain the results have hindered early diagnosis of the disease. Better testing with non-sputum samples, like blood, will be desirable and sustainable to diagnoses, which will inform therapy and reduce disease spread in communities.

A variety of methods have been employed for diagnosing tuberculosis, including chest X-ray, clinical symptoms, sputum smear testing, and tuberculin testing^8^. These methods have a range of disadvantages, such as difficulty collecting sputum from younger patients, time consumption, low performance, live mycobacteria tuberculosis is needed, sophisticated instruments that can only be managed by highly trained health care professionals, and therefore high cost^9^. No current diagnosis test is reliable and affordable enough, and existing tests in children and those with extra-pulmonary conditions are still unreliable^10^.

Due to these limitations in the various ways of tuberculosis diagnosis, especially with regards to pediatric and extra-pulmonary patients, there is the need to research alternative diagnostic methods that can aid diagnosis. There has been considerable scientific interest in alternative diagnostic tools for tuberculosis; mainly, blood transcriptional biomarkers are being considered.

Blood-based signatures are now a desirable choice since blood is accessible clinically for the reading of body immunology^11^; signatures of entire blood gene expression diagnosed with TB have been recognized in several works of literature^12–14^. Pathogens activate an immune-host reaction, and diagnostic signatures could be discovered by quantifying these responses. A standard method for establishing a biological signature is the use of values of DNA microarray gene expression, mostly acquired from blood samples of the host. Due to the relevance of developing new diagnostics methods for TB, a few blood-based (transcriptional) signatures have been proposed^14^.

The method of data classification using knowledge derived from documented historical data was one of the most widely researched subjects in statistics, data science, and computer sciences. Digital data and decision support systems are used to help medical professionals define the disease condition and help them evaluate a vast quantity of available data from previous cases and prescribe a suitable diagnostic based on values with different essential features. Data mining and machine learning methods have been applied to medical care in many ways, such as predicting the efficacy of surgical events, medical tests, medication, and clinical-diagnostic relations^1,2^.

In typical machine learning algorithms, the aim is to learn from training data on one hypothesis. However, the approaches used in an ensemble involves various learners that are termed base learner. Base learners are trained to solve similar problems. Thus, a collection of hypothesizes is constructed and being combined for a prediction^5^. A classifier that uses a small set of rules to classify examples from the future can lead to errors. An ensemble of classifiers is a group of classifiers that, in a certain way, incorporate individual choices in the classification of new examples. Several research results have shown that these multiple classifiers can boost the classification accuracy if properly combined during classification^2^. An ensemble of classifiers has proved to be a very efficient way of promoting classifying efficiency because unrelated errors created by a single classifier can be avoided. This paper enhances the existing weighted voting ensemble learning technique to aid in the development of a diagnostic model for the prediction of early tuberculosis infection.

## Related work

Considerable work efforts have been made to try to identify a signature that is transcriptional from patient blood so that we can effectively diagnose TB. However, a regular signature has remained evasive for heterogeneous populations^14^. Various kinds of deadly illnesses that could theoretically outspread to other areas of the body would be found. Therefore, predicting the existence of such phenomena is necessary in order to be able to prune the degree of the spread out. Investigating the attributes of genetics gives a deep instinct about the classification of diseases since they play an essential role in affecting exactly how an organism appears, acts, and survives in an atmosphere. Discovering abnormal genes family might be adequately modeled by utilizing statistical methods and machine learning approaches^15^. Several studies discovered alterations in transcriptome found in the host body in collaboration with TB infection, when likened with healthier means, people with LTBI, or any other ailments. Signatures have become increasingly concise as time passes, generating their near-patient translation in diagnostic assessments a lot more doable^16^.

Information provided by the gene expression from a microarray may act as a statistical tool for this calculation. Ragunthar et al.,^15^ treats the microarray as a recent leap in molecular biology, as it offers a framework for hybridizing the DNA samples and can be interpreted as values compatible with the level of gene expression the genome has. They propose a method for selecting a subset of services from the substantial amounts of trials from the gene expression profiles using the Boruta selection function algorithm, and they performed a comparative analysis with various supervised classification algorithms that were placed in place to classify the subset into a normal and deviant gene. The goal was to find the algorithm most appropriate for classifying the data of the gene expression. Therefore, a variety of abnormal genes may quickly be accelerated.

Warsinske et al.,^13^ performed a study that systematically contrasts 16 Host-response-based gene signatures for tuberculosis prognosis, 16 gene signatures when it comes to the medical diagnosis of ATB in comparison to different clinical circumstances is recovered from PubMed, each signature is applied with classification product expressed when looking at the corresponding initial publishing for the trademark, 3083 transcriptomes profiles obtained from 24 datasets containing whole bloodstream or peripheral mononuclear cell from 14 countries was gotten from GEO. From 9 datasets of people with a culture-confirmed medical ATB diagnosis, 11 signatures gave a weighted mean AUROC > 0.8 while two signatures got adjusted mean AUROC of 0.6. All but two signatures have a high negative value that is predictive > 98% at 2% prevalence). Caused by the research unearthed that host-response-based diagnostics could truthfully determine people with ATB and foresee those with a risk that is high of from LTBI to ATB previous to sputum transformation, a higher amount of family genes in a trademark failed to improve the accuracy of this signature. The Sweeney3 signature done robustly across all reviews, the effect supplies intense research for the possibility of host-response-based diagnostics and may feel in pursued medical execution.

A systematic analysis of comprehensive whole bloodstream transcriptional signatures for active tuberculosis was performed by Gupta et al.^16^ to compare the findings of methodically defined selection signatures for active tuberculosis and critically assessing the prospective importance of whole bloodstream transcriptional signatures as biomarkers for active tuberculosis. The study reveals, first and foremost, for a comparable symptom precision in the detection of active tuberculosis for eight transcription signatures such as a single transcript (BATF2) was found. Studies showed a near range of results, as in the UK and sub-Saharan Africa.

Taking into consideration the limitations because of the standard that is currently in the medical diagnosis of tuberculosis, Duffy et al.^11^ suggest blood-based signatures as a nice-looking alternative since bloodstream has a clinically easily readout available for this immunology county for the human body. The research dedicated to a comparison that is multinomial as opposed to the single binary review in consequent research. Multinomial signatures identified as signatures that predict more than two groups were trained simultaneously to design each patient's TB and HIV condition.

Their aim was to increase the prediction and generalization of the transcriptional signature for TB by explicitly training a multi-signature to foresee several HIV and TB conditions. Four previously available TB and HIV co-infection relating datasets were utilized, linear modelling of signature gene appearance is used to analytically identify genes as HIV-only, TB-only or merged HIV/ TB, six machine classifiers comprising of random forest, SVM, neural networks, K-nearest neighbor, were utilized to coach and confirm the dataset even though the predictive classifier performance was measured making use of leave-one-out cross-validation in the training set, numerous test sets making use of area beneath the ROC curve (AUC) and the abilities metric was shown. An improved consequence with AUC of 0.88 and a 10-gene trademark that discriminated against other non-TB infection from active TB states are acquired through the research.

Aided by the growing concern for reproducibility of biomarkers among researchers, especially in the field of bioinformatics, Bobak et al.^14^ discussed the reasons why results from homogeneous cohort researches are not generalizable for an inherently heterogeneous population. Within their efforts, they integrated four unique tuberculosis (TB) information cohorts to acquire typical differentially managed genes that could be utilized in identifying active from latent TB, some other disorders, and healthy controls. Twenty-five family genes had been chosen to utilize random forest, and an AUC of 0.89 was obtained from their training data, and 0.86 gotten in their test facts. It was discovered that 18 out of the 25 family genes were formerly involved in TB infection in the past separate research, which suggests that data integration can be an essential technique for growing microarray data reproducibility.

## Material and method

**Figure 1:**
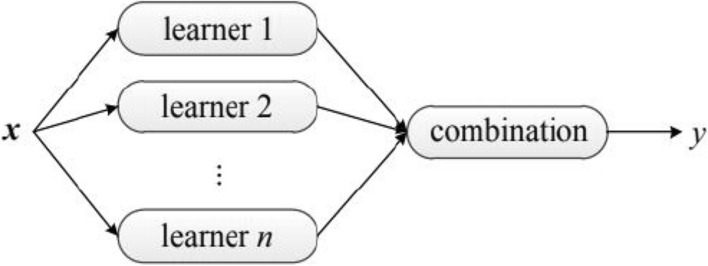
An ensemble architecture^5^.

Ensemble learning is also known as committee-based learning or multiple classification systems; a typical ensemble learning architecture is shown in Fig. 1. There exist two types of ensembles, those that use a single algorithm to construct homogeneous simple learners, leading to homogeneous ensembles, i.e., an ensemble of decision trees. The second type consists of the use of different learning algorithms to construct heterogeneous base learners, leading to heterogeneous ensembles, i.e., an ensemble of neural networks and SVMs^5^. The use of an ensemble often leads to a much stronger generalization than the use of a single learner. A significant advantage of ensemble methods is proven in its ability to improve feeble learners and turn them into strong learners with more accuracy.

Rather than using an algorithm to create a study model, ensemble methods are often used in order to build and integrate a range of classifiers, weak or strong, usually referred to as basic learners. The efficiency of an ensemble method is to carefully select both the base learners and the combined method for the final hypothesis^17^. In order to obtain stronger ensemble learners, every learner must make a significant number of possible predictions, and every model generated is unique from other models. The emphasis on relationships between variance, noise, bias, and covariance makes ensemble learners more efficient than base learners^18^. Technically, ensemble learning is mainly carried out in two steps: primary classifications for training and the selective combination of members' classifiers into a better classification^19^.

There are several appropriate and efficient ensemble methods, such as bagging, stacking, and boosting^19^, voting and averaging are common and widely used methods for combination considering the problem to be solved^17^.

i. *Bagging* Which is Bootstrap Aggregating, uses the base algorithm for bootstrapping samples from the training data. A bootstrap sample of n cases is formed by a random selection of n replacement cases. By calling a base learning algorithm, bagging trains several basic learners, each from a different bootstrap sample. A bootstrap sample is obtained by subsampling a replacement training dataset, where the sample size is the same as that of the training dataset^19^.
ii. Boosting is a method used to produce exact prediction rules by integrating many "weak" rules, which are only moderately accurate. The principal variation between several boosting algorithms is their method of weighing samples and hypotheses for training. The first member of the ensemble is created through the application of the base learning algorithm to the whole training set. Subsequent members of the ensemble are created by applying the base algorithm to the training set but with cases reweighted to put a higher weight on cases misclassified by existing members of the ensemble. AdaBoost is a typical example^19^.
iii. Stacking involves the generation of some first-level individual learners from the training dataset, using various learning algorithms. These individual learners are combined with a second-level learner called a meta learner.

The majority voting and weighted voting for classification are commonly used among the popular combination methods.

i. Simple majority voting is a decision rule which selects the highest number of correctly predicted values based on the predicted classes with the most vote.
ii. Majority voting; most votes do not require tuning parameters once a single classifier has been trained
iii. In weighted voting, voting weights should be different in each classification between different classes of results. For that particular class for which the classification is performing well, the weight should be high. Therefore, it is essential to identify the proper voting weights for each class per classifier^19^.

Methods like averaging take the sum of the performance of each classifier. They then average it to get the final prediction result in terms of weighted averaging weighted are assigned to the classifiers, and then the average is taken.

**Figure 2:**
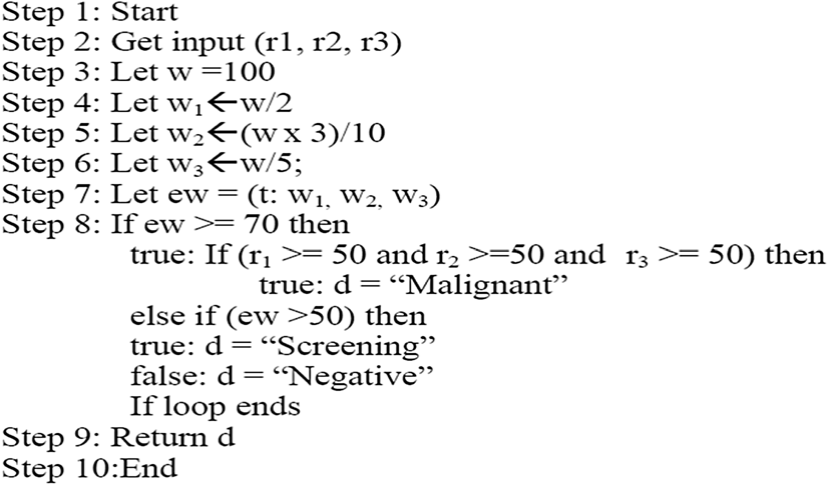
Weighted voting algorithm^21^.

**Figure 3:**
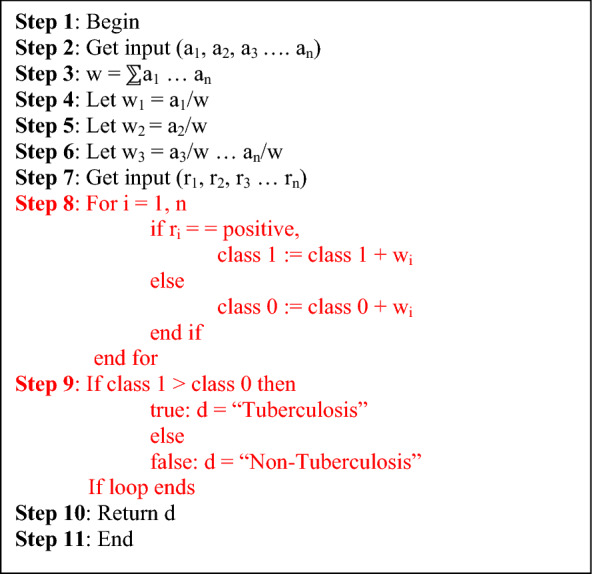
Extended weighted voting ensemble algorithm.

The use of ensemble learners has been seen to produce more encouraging results in several approaches and studies in which they have been utilized^3,15,19,20^. Devi and Audithan^21^ proposed extended weighted voting for the prediction of breast cancer; this algorithm is given in Fig. 2. The proposed algorithm is enhanced for the purposed of this study in the areas of the selection of weight for the various classifiers. Also, the final classification part of the algorithm is likewise modified in the proposed weighted voting algorithm. The proposed algorithm is given in Fig. 3. In the existing algorithm, Devi et al.^21^ employed the use of three machine learning algorithms KNN, SVM and Naïve Bayes. They weights of each classifiers were chosen arbitrary to obtain a total 100 and then the probability of classifiation was used to determine the final ensemble result. This is seen in the algorithm below.

The reason for employing ensemble methods in building a model is to enhance the overall performance of the model and to minimize the error rate that can be caused by using a single classifier. The weighted voting method assigns various weights to the classifiers based on specific criteria and takes a vote of the classifiers based on the weight. In this work, the weight of each classifier would be chosen based on the performance accuracy of the classifier based on the training set. The formulae in Eq. (1) would be used in assigning the weight for each classifier.

1 $${W}_{c}= \frac{{A}_{c}}{\sum {A}_{n}}$$

where W_c_ is the weight of classifier, A_c_ is the accuracy of the classifier, and A_n_ is the summation of the accuracy of all classifiers used.

After getting the calculated weight for each of the classifier, the classification result that has the highest vote is finalized as the final result for the ensemble. This enhanced algorithm defers from the algorithm capture in Devi et al.^21^ in the area of choosing the weight for each classifier and also in the area of the method for final classification. The algorithm utilized the exact classification of a sample by the classifier as either TB or Not while the existing algorithm used the probability the the sample was classified as either TB or Not.

The enhanced weighted voting algorithm that would be used as the ensemble method is given below:

### Extended weighted voting ensemble (EWVE) algorithm

a = accuracy of classifiers; w = sum of all classifier’s accuracy; r = prediction of classifiers; class 1 = positive; class 0 = negative.

**Figure 4:**
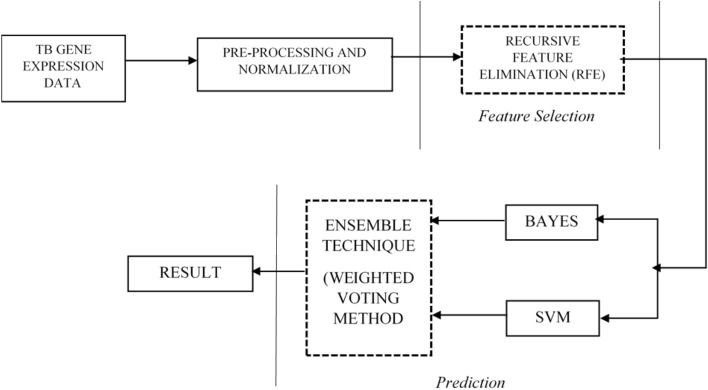
Model architecture for EWVA.


The model architecture shown in Fig. 4 below was used as the architectural guide in the development of the model. The steps were followed sequentially to obtained the desired result of the research.

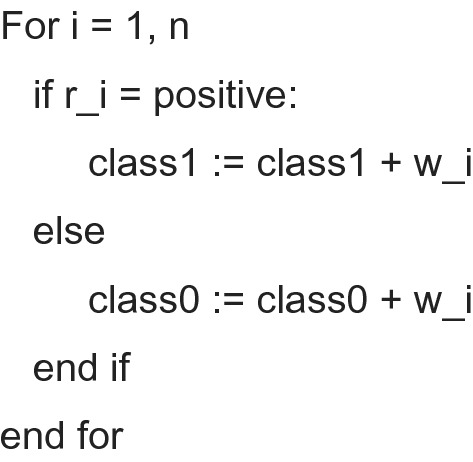


The tuberculosis gene expression dataset obtained from the NCBI GEO site was made up of 48,803 genes against 498 samples. These samples were grouped and classified into patients with PTB (Active TB) comprising of 103 samples and other disease made up of 395 samples. The number of genes must be reduced to select only the relevant genes that would be used for developing the model. This reduction was made by applying dimensionality reduction and feature selection processes to the dataset using principal component analysis (PCA) and recursive feature selection cross-validation (RFE-CV) methods. Before the feature reduction methods was applied; The probe id was matched to their corresponding gene symbols, and those probe ids that had no gene symbols were removed from the dataset, reducing the number of genes to 36,157 genes. Duplicated genes were also removed and the number of genes dropped to 25,159 genes, which was then used for the dimensionality and feature selection. Now all duplicated genes have been mapped together as just one gene symbol.

### Dimensionality reduction using PCA

The method was ran on the dataset made up of 456 observations (Samples) and 25,159 variables or features (Genes). The data was transposed before running the method; after the completion of the process, the essential variables generated by the method was 457 PC (principal components), which are the genes. After the dimensionality reduction, the genes are still much and may not give optimal results if used to develop the model for classification, hence the need sfor a feature selection process.

### Feature selection using recursive feature elimination-cross validation method (RFE-CV)

**Figure 5:**
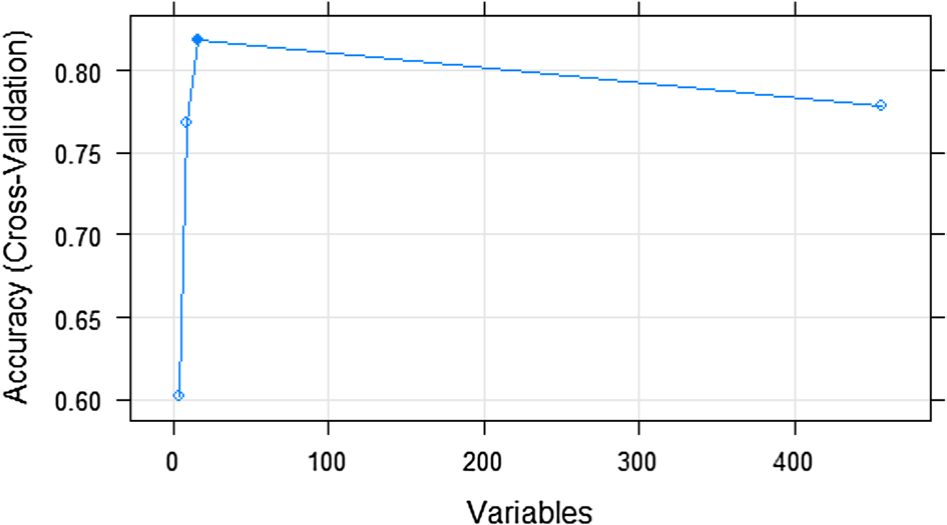
Plot of RFE-CV result.

The RFE method was chosen because it is a simple feature selection method and does not take much computational time. The RFE method was carried out on the reduced dataset from the PCA preprocessing stage, which comprised of 456 observations (Samples) and 457 variables (principal components /genes). The number of cross-validation folds used in the process was ten folds; after the completion of the process, a set of 16 variables with an accuracy of 0.81 was selected as the important variables from the dataset. Figure 5 shows the feature selection plot of the result.

The dataset was randomly divided into two: the train set, which was made up of 80% of the dataset, and the test set comprised of the remaining 20%. The dataset used was inbalanced as the ratio of sample with TB was not equal to that of samples without TB, hence the use of other performance matrics senitivity and specificity to ascertain the performance of the built model.

## Result and discussion

The models used in this work were developed and trained using a tuberculosis gene expression dataset obtained from the NCBI GEO website. The dataset comprised of different genes obtained from patients with tuberculosis and those without tuberculosis. The dataset was randomly divided into two: the train set, which was made up of 80% of the data, and the test set, which comprised of the remaining 20%. The train set was used in developing and training the models, while the test set was used to ascertain the performance of the trained model using different performance metrics, which include accuracy, specificity, and sensitivity.

The algorithms used for the model development were support vector machine (SVM) and Naïve Bayes (NB) algorithms. The ensemble technique was finally used to combine these models together to improve the performance accuracy of the classification model. The weighted voting method was used as the ensemble technique in this work. The results of the performances of the individual algorithms, the existing weighted voting technique, and the improved weighted voting technique are shown below. Table 1 gives a summary of the performance metrics used in the evaluation of the model.

**Table 1:** Performance metrics.

Metrics	SVM	NB	Ensemble (existing)	Ensemble (improved)
Accuracy	0.92	0.87	0.90	0.95
Specificity	0.66	0.88	0.50	0.95
Sensitivity	0.98	0.87	1.00	0.94

The result of the developed model for the diagnosis of tuberculosis shows that the model is effective and can support medical practitioners in the early diagnosis of the disease. The result in Table 1 shows the performance accuracies, sensitivity, and specificity of the individual classifiers used in the work. In terms of performance based on sensitivity which shows how the classifiers performed in measuring the number of correctly classified positive instances against the number of supposed positive instances, and specificity which shows measures of the correctly classified negative instances, the confusion matrix was used to show this result from each classifier explicitly. The figures and table below shows the result per classifier used in the study.

**Figure 6:**
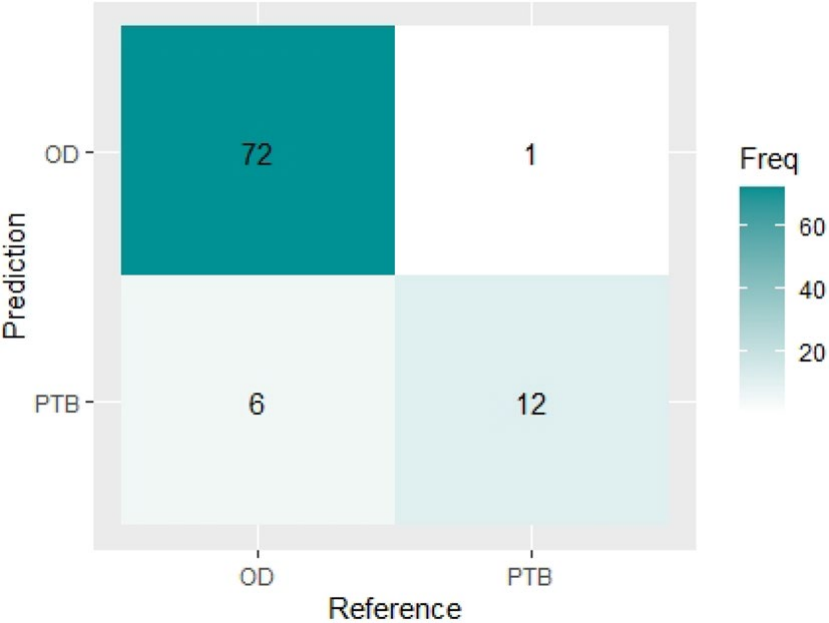
Visual representation of SVM classifier’s confusion matrix.

Table 2 gives the result of the confusion matrix of the classification model using the Support vector machine on the test set. It shows that the trained model accurately classified 72 patients with other diseases (OD) correctly and 12 patients with tuberculosis (PTB) correctly. Therefore, out of the 91 number of samples in the test set, 84 (72 + 12) were classified correctly, while seven samples were classified wrongly (1 OD classified as PTB and 6 PTB as OD) by the model. Figure 6 shows the visual representation of the SVM classifier’s confusion matrix report.

**Table 2:** Confusion matrix report for SVM.

	Class predicted by the model		
	OD	PTB	
**Actual classes**			
OD	72	6	78
PTB	1	12	13
	73	18	91

**Figure 7:**
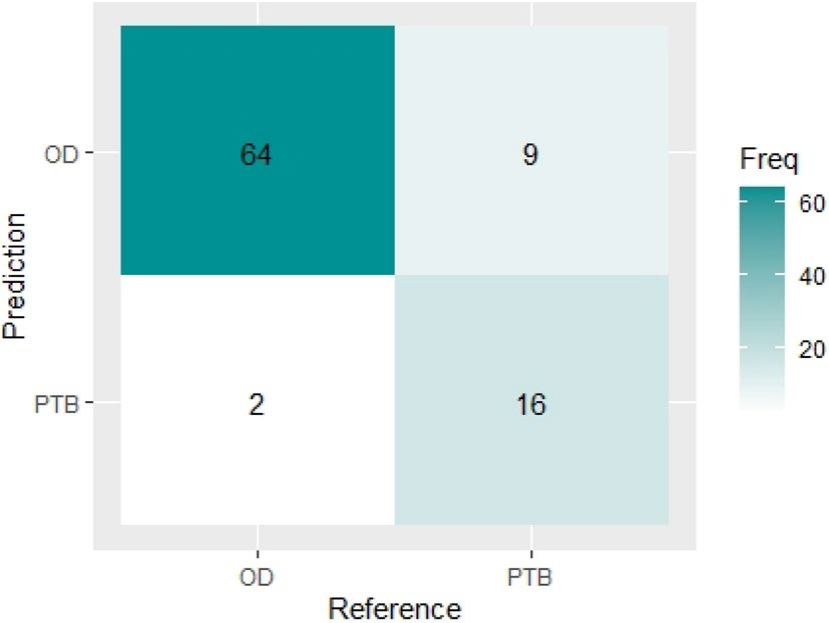
Visual representation of NB classifier’s confusion matrix.

Table 3 shows the result of the confusion matrix of the classification model using the Naïve Bayes Algorithm on the test set. It shows that the trained model accurately classified 64 patients with other diseases (OD) correctly and 16 patients with tuberculosis (PTB) correctly. Therefore, out of the 91 number of samples in the test set, 80 (64 + 16) were classified correctly, while 11 samples were classified wrongly (9 OD classified as PTB and 2 PTB as OD) by the model. Figure 7 shows the visual representation of the NB classifier’s confusion matrix report.

**Table 3:** Confusion matrix report for NB.

	Class predicted by the model		
	OD	PTB	
**Actual classes**			
OD	64	2	66
PTB	9	16	25
	73	18	91

**Figure 8:**
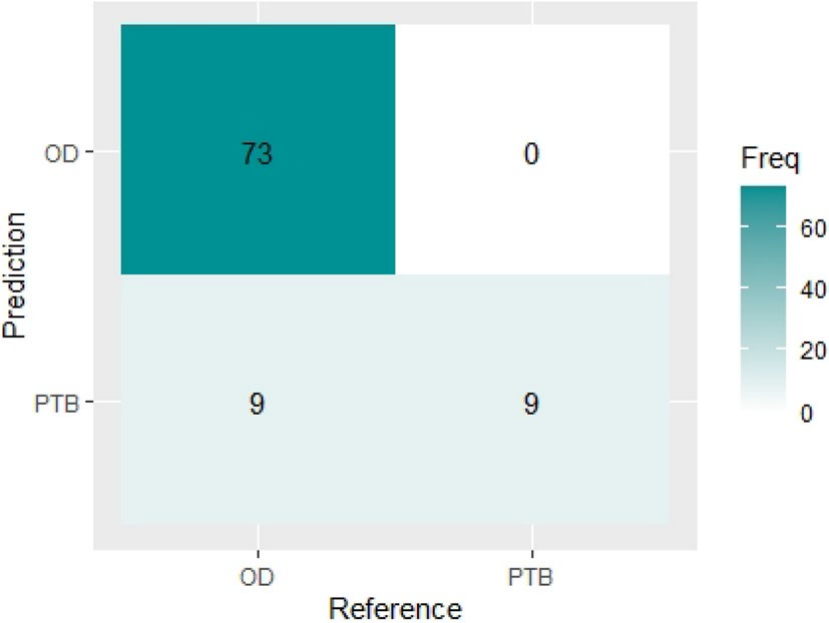
Visual representation of existing weighted voting ensemble method confusion matrix.

Table 4 shows the result of the confusion matrix of the classification model using the existing weighted voting ensemble method on the test set. It shows that the trained model accurately classified all 73 patients with other diseases (OD) correctly and nine patients with tuberculosis (PTB) correctly. Therefore, out of the 91 number of samples in the test set, 82 (73 + 9) were classified correctly, while 9 samples were classified wrongly (9 PTB classified as OD) by the model. Figure 8 shows the visual representation of the existing weighted voting ensemble confusion matrix report.

**Table 4:** Confusion matrix report for existing weighted voting ensemble method.

	Class predicted by the model		
	OD	PTB	
**Actual classes**			
OD	73	9	82
PTB	0	9	9
	73	18	91

**Figure 9:**
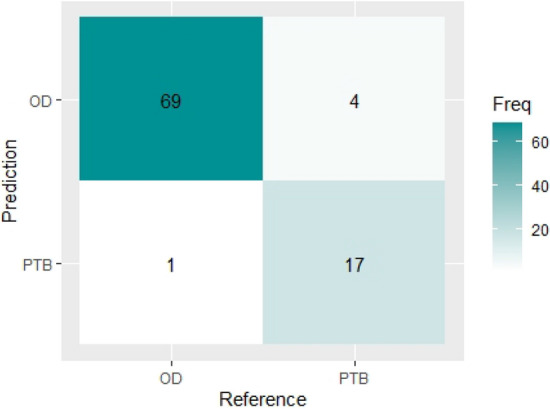
Visual representation of improved weighted voting ensemble method confusion matrix.

The result of the confusion matrix of the classification model using the improved weighted voting ensemble method on the test set is seen in Table 5 below. It shows that the trained model accurately classified 69 patients with other diseases (OD) correctly and 17 patients with tuberculosis (PTB) correctly. Therefore, out of the 91 number of samples in the test set, 86 (69 + 17) were classified correctly, while 5 samples were classified wrongly (4 OD classified as PTB and 1 PTB as OD) by the model. Figure 9 shows the visual representation of the improved weighted voting ensemble confusion matrix report.

**Table 5:** Confusion matrix report for improved weighted voting ensemble method.

	Class predicted by the model		
	OD	PTB	
**Actual classes**			
OD	69	1	70
PTB	4	17	21
	73	18	91

These individual classifiers have also been used in several studies in developing a model for the diagnosis of tuberculosis as it relates to having a transcriptional signature. In the work of Roe et al.^22^, they used a support vector machine (SVM) with feature selection to develop discriminating models with the transcriptomic data from patients with and without active TB, the accuracy of their developed model was 0.93. Feature selection is important when dealing with transcriptomic data (which is also gene expression data). Deng et al.^23^ also used an SVM classifier to develop a model and obtain an accuracy of 0.911 from the model. For Bobak^14^, they used a random forest (RF) algorithm to develop a classification model for tuberculosis diagnosis. The accuracy of their developed model, was 0.89 for the training set and 0.86 for the test set. However, this study integrated four different datasets together, and this was used as input; this may, in turn, could have an impact on the model development, but the result of the developed model is still good. SVM, KNN, and RF were used for the same purpose of developing a model for gene expression data classification in the work of^15^. These results can be improved on and that entails apply the ensemble method. This was also given as a recommendation in this study. In this study, using SVM and NB classifiers we obtained the accuracies of 0.92 and 0.87 respectively,which result is also in agreement and better than some result obtained in some of the studies discussed above.

In this work, we went further to apply the ensemble learning technique to improve the performance of the single classifiers used. The ensemble learning technique involves combining the results of different or same classifiers to get a result. The main reason why the ensemble learning technique has been used is to improve the performance classification model been built by integrating the results of single classifiers and aggregating their results to obtain the final result. As at the time this work was done, there has been no implementation of using the ensemble learning method in relation to transcriptional signatures for tuberculosis diagnosis. For this reason, the existing algorithm adapted from^21^ was implemented for a breast cancer study; however, this algorithm was reimplemented and used for this work.

The improvement that was made on the existing algorithm was in choosing the weight of the single classifiers: this study made use of the accuracy of each classifier to calculate the respective weights. For the combination of the final result, the existing algorithm made use of the probability of each sample in the class. In contrast, the improved algorithm used just the class the sample was classified as and chose the class with the highest number. The result of the implementation of these algorithms showed that the improved algorithm performed better than the existing algorithm with an accuracy of 0.95 as against an accuracy of 0.90 obtained from the existing algorithm. This result obtained from the ensemble method also shows that the ensemble learning technique also improves the accuracy of individual classifiers as it was also higher than the accuracy of the individual classifiers. Hence, this result proves that the ensemble learning technique is an efficient method of improving a classification model's performance.

The ensemble algorithm was used on another tuberculosis dataset, the preprocessing and feature selection methods were the same as what was used on the initial dataset, PCA was used for dimensionality reduction and the RFE-CV was used in selecting the final features used in the model development. Again the ensemble technique was seen to produce a good performance report in terms of accuracy of the model classification. Table 6 below gives the peformance summary of the dataset in terms of classifying TB paitents from Non-TB patients.

**Table 6:** Performance metrics.

Metrics	SVM	NB	Ensemble (existing)	Ensemble (improved)
Accuracy	0.95	0.96	0.92	0.96
Specificity	0.60	0.70	0.40	0.70
Sensitivity	1.00	1.00	1.00	1.00

From the table above, it can be seen that all models performed optimally in classifying the negative class (Non-TB) correctly while variations can be seen in the specificity result, but the improved algorithm tends to produce the best result in terms of specificity alongside Naïve Bayes classifier compared to others. It is also seen that the improved ensemble algorithm performed better than the existing ensemble algorithm for this dataset; hence this shows a good performance result for the algorithm. More classification models can be added in future study, and then the ensemble technique can be incorporated to obtain an optimal result from those models.

Generally, the ensemble technique employed for this study has not been used in any study related to TB diagnosis. Most studies have used one or more classifiers to develop their models and, in turn, make a comparison between the individual classifiers to identify the classifier that yielded the best performance accuracy. This study went a step further to implement the ensemble method using the single classifiers. The performance accuracy obtained from the individual classifiers is similar to those obtained in previous studies. The ensemble method used obtained a better result, which shows the advantage of using the ensemble method. As a recommendation for future work, the model can be implemented to provide medical practitioners an interaction interface.

The novelty of this work originates in the strategy for improving the existing ensemble algorithm. This was achieved by calculating the individual weights of each classifier instead of using arbitrary weights as in the existing algorithm. Our new approach has enabled the easy assignment of the final result given to the classifier with the highest weight based on the classification runs. It creates more hope that tuberculosis which has is causing more death, is gradually getting attention for innovative diagnosis and gives more credence to the improved classification result.

In most previous studies^4,14,24^, single machine learning algorithms have been used for classification, and in cases where more than one algorithm is used, the emphasis is on comparison to determine which of the algorithm had a better performance. However, in this work, we introduced the use of the ensemble technique to combine more than one machine learning algorithms to produce an optimal result that shows performance is better using an ensemble for classification than using a single classifier. This approach has not been used in any study that relates to tuberculosis classification using gene expression dataset as at the time this research was carried out.

## Conclusion and future work

Health-related issues are always promoted and need to be attended to in the fastest and easiest way possible. The need to improve medical diagnosis and treatment methods cannot be over-emphasized, considering the cost of providing these services knowing that every individual's health is of utmost importance. Tuberculosis disease has been a significant life-threatening disease as it can be easily spread through the air, and the mortality rate has still been on the high side on an annual basis as recorded by the WHO report. The current methods of diagnosing TB are not utterly inadequate. However, the limitations surrounding these methods, like the cost of diagnosis, requiring sputum to carry out the test, and the time taken for the results to be obtained, have prompted more straightforward methods and provides time results.

Transcriptional signatures obtained from the blood have become a desirable alternative. The body changes can be reflected by genes obtained easily by studying the blood samples, and blood is also readily available and can be easily obtained from patients. In this work, these transcriptional signatures were used to develop a classification model that would aid TB's diagnosis in a more efficient and fast way. The developed model made use of gene expression data; the transcriptional signatures were obtained from this data. The model was trained using 80% of the dataset, while 20% was used in testing the performance accuracy of the developed model. Two classifiers SVM, and NB, was used in the development of the model, and an improved weighted voting ensemble method was used to finalize the model development to improve the classification accuracy of the individual classifier; this gave an accuracy of 0.95, which was higher than the accuracies obtained from the single classifiers. This implies that using the ensemble techniques improves the performance accuracy of a classification model developed using individual classifiers when combined. This gives more credence to the model been developed to solve a particular classification issue. It is worthy to note that efforts have been made to develop medical diagnosis tools^24^ especially for TB infections^25^ this enhanced Weighed Voting Ensembled will further contribute to the machine learning solutions in TB diagnosis.

## Data Availability

The tuberculosis gene expression data that was used in this study are available in the Gene expression omnibus (GEO) database, https://www.ncbi.nlm.nih.gov/geo/query/acc.cgi?acc=GSE19491https://www.ncbi.nlm.nih.gov/geo/query/acc.cgi?acc=GSE42834.
